# Nontuberculous Mycobacterial Pulmonary Disease from *Mycobacterium hassiacum*, Austria

**DOI:** 10.3201/eid2611.191718

**Published:** 2020-11

**Authors:** Helmut J.F. Salzer, Bakari Chitechi, Doris Hillemann, Michael Mandl, Christian Paar, Monika Mitterhumer, Bernd Lamprecht, Florian P. Maurer

**Affiliations:** Kepler University Hospital, Linz, Austria (H.J.F. Salzer, B. Chitechi, C. Paar, M. Mitterhumer, M. Mandl, B. Lamprecht);; National and WHO Supranational Reference Centre for Mycobacteria–Borstel, Borstel, Germany (D. Hillemann, F.P. Maurer)

**Keywords:** 16S rRNA, antimycobacterials, Austria, bacteria, Mycobacterium hassiacum, nontuberculous mycobacterial pulmonary disease, NTM-PD, tuberculosis and other mycobacteria

## Abstract

The clinical relevance of newly described nontuberculous mycobacteria is often unclear. We report a case of pulmonary infection caused by *Mycobacterium hassiacum* in an immunocompetent patient in Austria who had chronic obstructive pulmonary disease. Antimicrobial drug susceptibility testing showed low MICs for macrolides, aminoglycosides, fluoroquinolones, tetracyclines, imipenem, and linezolid.

Currently, >170 species of nontuberculous mycobacteria (NTM) are recognized (*1*), most considered nonpathogenic to humans. However, some NTM can cause severe pulmonary disease. We recently observed a case of NTM pulmonary disease (NTM-PD) in Austria that was caused by *Mycobacterium hassiacum*. 

In January 2019, a 62-year-old man was admitted to the outpatient clinic at Kepler University Hospital in Linz, Austria, having had dry cough and progressive dyspnea for several months. No weight loss or night sweats were reported. He had a medical history of chronic obstructive pulmonary disease with severe emphysema because of cigarette smoking. The patient had no history of tuberculosis or NTM-PD and was unaware of any contact with persons with mycobacterial infections. 

Chest radiograph showed new consolidations in the right and left upper lung lobes compared with images obtained 1 year before. We performed a high-resolution computed tomography scan of the chest and an 18F-fluorodeoxyglucose positron emission tomography (18F-FDG PET) scan that indicated metabolic activity consistent with an inflammatory process (Figure). In addition, results of tests for serum lung cancer biomarkers, including CA 19-9, CEA (carcinoembryonic antigen), CYFRA 21-1, and NSE (neuron-specific enolase), and for HIV-1 and HIV-2 were negative. Transbronchial catheter aspiration from the posterior lung segment of the right upper lung lobe showed no microscopic evidence of bacteria, acid-fast bacilli, or fungi. The result from a PCR assay (*Mycobacterium tuberculosis* PCR kit; Geneproof, https://www.geneproof.com) for *M. tuberculosis* complex DNA was negative. Results from bacterial and fungal cultures were unremarkable. Because the patient was at high risk for developing pneumothorax due to severe emphysema, no transbronchial biopsy was taken during initial bronchoscopy. 

**Figure:**
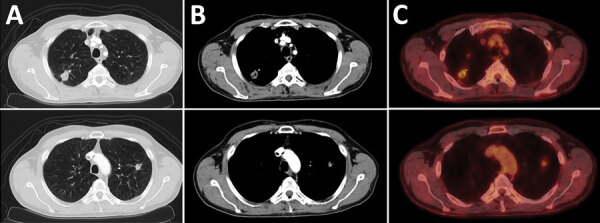
High-resolution computed tomography and 18F-fluorodeoxyglucose positron emission tomography scans of the chest showing pulmonary lesions caused by *Mycobacterium hassiacum* in a 62-year-old man, Austria. A and B) Computed tomography scans of the chest showing a subpleural thick-walled cavitary lesion in the posterior segment of the right upper lung lobe with associated pleural thickening and a smaller adjacent partly calcified solitary nodule. Another solid nodule of 13 mm diameter was found in the left upper lung lobe. C) Positron emission tomography scan showing a tracer uptake in both lesions with a standardized uptake values of 5 (top image) and 1.9 (bottom image).

Mycobacterial culture results of the transbronchial catheter aspirate were negative after 8 weeks of incubation. Consequently, we performed a computed tomography–guided needle biopsy of the cavitary lesion in the right upper lung lobe. Histological results showed granulomatous inflammation with focal eosinophilic necrosis. We detected no atypical cells, acid-fast bacilli, or fungal hyphae and subsequent immunohistochemistry testing revealed no evidence of malignant disease. 

Results of a PCR of formalin-fixed, paraffin-embedded lung tissue revealed no DNA of *M. tuberculosis* (MTB Nested ACE Detection Kit, Seegene, http://www.seegene.com) or of 13 different NTM species (MYCO Direct 1.7 LCD-Array Kit; Chipron, https://www.chipron.com). However, native lung tissue was used to set up both liquid (MGIT 960 system, Becton Dickinson, https://www.bd.com) and solid mycobacterial cultures (Löwenstein-Jensen and Middlebrook agar), which showed growth after 21 days of incubation at 37°C. 16S rRNA gene sequence analysis identified *M. hassiacum* (GenBank accession no. AF547933.1). 

We analyzed the samples by DNA sequencing of the first 500 bp of the 5¢ region of the 16S rRNA gene (*2*,*3*). The analyzed DNA fragment showed 500-bp identity with the *M. hassiacum* type strain 3849 16S ribosomal RNA (sequence identification NR_026011.1). More than 25 mismatches were found with the type strains of *M. thermoresistibile* strain NCTC10409, *M. goodii* strain ATCC 700504, and *M. celeriflavum* strain AFPC-000207. Antimicrobial susceptibility testing by broth microdilution method according to Clinical and Laboratory Standards Institute guidelines (*4*,*5*) showed low MICs for clarithromycin (≤0.06 μg/mL), amikacin (≤1 μg/mL), tobramycin (≤1 μg/mL), linezolid (≤1.0 μg/mL), moxifloxacin (≤0.25 μg/mL), ciprofloxacin (0.25 μg/mL), doxycycline (≤0.12 μg/mL), minocycline (≤1 μg/mL), tigecycline (0.25 μg/mL), imipenem (≤2.0 μg/mL), and trimethoprim/sulfamethoxazole (4.75 μg/mL) (*4*,*5*). Comparably high MIC values were observed for cefoxitin (32 μg/mL), ceftriaxone (>64 μg/mL), and cefepime (>32 μg/mL). Antimycobacterial treatment, including clarithromycin (500 mg orally 2×/d), moxifloxacin (400 mg orally 1×/d), and minocycline (100 mg orally 2×/d), was initiated. On first follow-up visit, the patient indicated that the treatment had been well tolerated. 

Since *M. hassiacum* was described as a new species in 1997, 3 cases of suspected infections caused by *M. hassiacum* have been reported in the medical literature (*6*). So far, *M. hassiacum* has been reported as the causative agent for peritonitis and cystitis (*7*,*8*). In addition, *M. hassiacum* was recently isolated from a respiratory sample in a patient in Germany with exacerbation of chronic obstructive pulmonary disease (*9*). However, that patient likely did not have NTM-PD because *M. hassiacum* was isolated in only 1 of 3 sputum samples, he showed no NTM-specific radiological abnormalities in a chest radiograph, and his clinical condition improved rapidly without any antimycobacterial treatment. 

In contrast, the patient we report fulfills 3 diagnostic criteria of NTM-PD: 1) an NTM-specific radiologic pattern characterized by new nodules in both upper lung lobes in a high-resolution computed tomography scan of the chest, 2) a positive culture result from a sterile computed tomography–guided needle biopsy, as well as typical mycobacterial histopathologic features including granulomatous inflammation, and 3) exclusion of other disorders including pulmonary tuberculosis or lung cancer (*10*). 

In conclusion, detection of *M. hassiacum* in patients fulfilling the criteria for NTM-PD should be considered a potentially relevant finding. Further studies are needed to evaluate the precise role of *M. hassiacum* as an emerging mycobacterial pathogen.
